# Reaching Consensus by Allowing Moments of Indecision

**DOI:** 10.1038/srep14839

**Published:** 2015-10-06

**Authors:** A. Svenkeson, A. Swami

**Affiliations:** 1 Army Research Laboratory, 2800 Powder Mill Road, Adelphi, MD 20783, USA

## Abstract

Group decision-making processes often turn into a drawn out and costly battle between two opposing subgroups. Using analytical arguments based on a master equation description of the opinion dynamics occurring in a three-state model of cooperatively interacting units, we show how the capability of a social group to reach consensus can be enhanced when there is an intermediate state for indecisive individuals to pass through. The time spent in the intermediate state must be relatively short compared to that of the two polar states in order to create the beneficial effect. Furthermore, the cooperation between individuals must not be too low, as the benefit to consensus is possible only when the cooperation level exceeds a specific threshold. We also discuss how zealots, agents that remain in one state forever, can affect the consensus among the rest of the population by counteracting the benefit of the intermediate state or making it virtually impossible for an opposition to form.

Master equations describing how the probabilities or populations of different states change over time have provided a universal framework for understanding an increasingly diverse number of phenomena lying outside the realm of traditional physics, including language evolution^1^,^2^, religious affiliation^3^, epidemic outbreaks, and the opinion dynamics of social groups^4^. The latter field has seen particularly rapid growth due to the popularity of online social networks. With relevance to elections, protests, conflict resolution, financial planning, and more, a central question of researchers studying opinion dynamics is the following: “What conditions are required for a group of interacting individuals to be able to reach a consensus?”

A different but related question, “How do macroscopic domains of aligned spins form in ferromagnetic materials when interactions between spins are local?”, was studied by the German physicists Ising and Lenz nearly a century ago^5^,^6^. The Ising model is now widely adopted to introduce concepts related to phase transitions and critical phenomena^7^. Although it was originally proposed to explain ferromagnetism, its simplicity allowed for drawing a connection to opinion dynamics^8^,^9^,^10^. Instead of magnetic moments, the units in the model are agents who decide between two options, and it is assumed that each agent is biased toward the majority opinion of its neighbors. Cooperative interactions lead to the emergence of global order, or consensus, when the interaction strength surpasses a critical value.

The social network structure determines the critical point at which consensus is possible. For example, less cooperation is required to reach a majority in a fully connected network compared to a two-dimensional lattice where interactions are limited to the four nearest neighbors of an agent. Ising dynamics have been studied on many network structures (random^11^, small world^12^,^13^,^14^, scale-free^15^) in order to categorize their respective critical points, if a phase transition still exists. Herein we take a different approach. Rather than focusing on the network structure, we are concerned with the cooperative dynamics that lead to the phase transition. To that end we study a fully connected network and extend the decision making model^16^,^17^,^18^, an Ising-like model similar to that of Weidlich’s pioneering work^9^,^10^,^19^, to include an intermediate third state. To change opinions an agent must first pass through the intermediate state, signifying temporary indecision.

The role of an intermediate state in opinion dynamics has been considered before in different forms: centrists in the voter model^20^,^21^ or similarly the AB model^22^, the binary naming game^23^, and recently in a model where each agent has a conviction that varies over time^24^. However, by including the intermediate state in an Ising-like model we gain a better understanding about the dynamical origin of the critical point associated to a second order phase transition. Namely, when allowing a variable transition rate for agents to leave the intermediate state we find that the location of the critical point is no longer fixed solely by the network structure. The less time the agents spend in the intermediate state, the easier it is for a majority opinion to form. As a result consensus can be reached with considerably less cooperative effort than is required in the case where agents are forced to choose between two polar opinions.

For clarity, we note that in the opinion dynamics literature consensus is often defined as an absorbing state where all the individuals share the same opinion. This is not the definition we adopt. In fact the Ising-like dynamics of the model we consider all but rules out this type of unanimous consensus, which resembles lowering the temperature of the system to absolute zero where fluctuations have disappeared. Instead, we are more interested in the capability of the group to form a nonvanishing majority opinion in the presence of individual fluctuations. Consensus is thus tied to the phase transition properties of the model. In the disordered regime, the majority opinion vanishes and we say that consensus does not exist. In the ordered regime, we say that consensus exists.

The influence of zealots on opinion formation among a population has proved to be significant, as a small number of individuals with a fixed opinion are capable of swaying the consensus their way. Zealots have been studied in a variety of cases, including the voter^25^,^26^ and similar models^27^, the binary naming game^28^,^29^,^30^, the decision making model^17^, evolutionary game theory^31^,^32^,^33^, and coevolving networks^34^. In our model we consider the general scenario where a fraction of zealots may be present in each of the three states. If the zealots populate the intermediate state, the rest of the agents, free to change opinion, spend more time without making a decision and the capability of the group to form a majority opinion can be lost. If the zealots favor one opinion over the other, a highly cooperative effort may be needed to form an opposition.

## Results

We start with a description of the model. At a given instant in time, an individual *i* of the group can be found in one of the three states


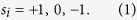


For example the state *s*_*i*_ = +1 may correspond to the opinion *yes*, *s*_*i*_ = −1 to the opinion *no*, and *s*_*i*_ = 0 to *undecided*. In the calculations that follow we label the state +1 with *x*, the state 0 with *y*, and the state −1 with *z*. The state of an individual is allowed to fluctuate over time between the three options. Analogous to magnetization in the Ising model, the mean field of the population,


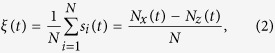


measures the polarization toward the global opinion of either *yes* (*ξ* = +1) or *no* (*ξ* = −1). *N* is the total number of individuals, while *N*_*x*_ and *N*_*z*_ are the number of individuals in the respective states *x* and *z*. The undecided individuals do not contribute to the mean field.

We are primarily concerned with the idealized scenario where the network of individuals is fully connected, and the number of individuals is infinite. When this is the case, the set of master equations


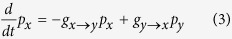



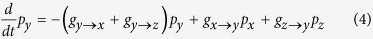



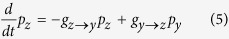


describes the dynamical behavior of the system, with *p*_*x*_, *p*_*y*_, *p*_*z*_ the probability for an individual to be found in the state *x*, *y*, or *z*, respectively. Equivalently, *p*_*x*_, *p*_*y*_, and *p*_*z*_ represent the fraction of the population in these respective states. The variable *g*_*x*→*y*_ signifies the rate at which an individual makes a transition from state *x* to state *y*. Similarly, this is true for transitions between any two states. Note that *g*_*x*→*z*_ = *g*_*z*→*x*_ = 0, meaning an individual cannot transition directly from *x* to *z* or vice versa, but instead must pass through the intermediate state *y*, representing the temporary indecision naturally associated with changing an opinion.

Moving away from the idealization of an infinitely large and well-mixed population is necessary for modeling real-world phenomena. Although we restrict our study to the fully connected network, it is possible to consider populations structured according to a specific network topology. In that case the transition rates would vary from one individual to the next depending on the states of the neighbors it is connected to, and the probabilities in Eqs. (3) (4) and (5) would correspond to a Gibbs ensemble of social networks. Lifting the infinite *N* restriction is also possible. The result would be stochastic master equations, with fluctuations having an intensity inversely proportional to *N*, if *N* ≫ 1. For more detail on the Langevin-like formalism useful for approximating the mean field behavior of a finite-size network see for instance ref. 35, or alternatively ref. 36.

To complete the definition of the model we must specify each of the transition rates. This is a subjective process. For example, we could choose transition rates *g*_*x*→*y*_ = *p*_*z*_, *g*_*y*→*x*_ = *p*_*x*_ + *p*_*y*_, *g*_*y*→*z*_ = *p*_*z*_ + *p*_*y*_, and *g*_*z*→*y*_ = *p*_*x*_ that lead us to recover the master equations known to describe the binary naming game^2^,^30^,^37^. Instead, we select Arrhenius rates:


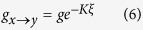



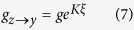


for transitions into the middle state, and


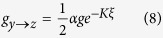



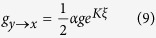


for transitions out of the middle state. *K* is the cooperation level between individuals, and *g* is the base transition rate that occurs when individuals do not interact (*K* = 0). Note that the parameter *α* is included in Eqs. (8) and (9) in order to allow a possible time scale separation between transitions into and out of the intermediate state. The factor of 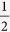 is explicitly written having in mind that, during any given time step in the numerical simulation of the model, half of the individuals in the middle state will attempt to transition to *x*, and the other half to *z* (see the methods section for more details). This way the transition rates are “normalized” by the number of allowed transitions from each state, meaning that in the absence of interactions, and assuming *α* = 1, the average number of individuals occupying each state is proportional to the number of possible transitions out of that state, 
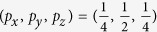
. Without the normalization the population would be divided equally among each state, 
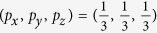
.

The cooperative nature of the social opinion model is revealed by examining Eqs. (6) (7) (8) and (9), where we set the condition *K* ≥ 0. When there are more individuals in the state *x* than there are in *z*, *ξ* > 0 and the transition rate from *x* to *y* is less than the base rate *g*, meaning that individuals in *x* tend to hold that opinion longer than they normally would. On the other hand, individuals not agreeing with the majority opinion tend to transition faster than normal. By making transitions faster or slower in this manner, an individual tends to mimic the majority opinion of the group.

### Phase transition behavior

The underlying purpose for choosing Eqs. (6) (7) (8) and (9) is to examine the feasibility of phase transition behavior, knowing that a phase transition exists for similar rates in the two-state decision making model. One advantage of this choice of transition rates is the presence of a control parameter *K* that defines the cooperation strength between individuals. *K* is analogous to the inverse of temperature in the Ising model. Starting from a group of independent individuals (*K* = 0) and then increasing the cooperation level *K*, a phase transition from a disordered, uncooperative population to an ordered, cooperative population occurs. In the two-state decision making model, this transition happens at the critical value *K*_*c*_ = 1 for a fully connected network^38^, as in the Ising model^7^. We are interested in finding out the critical value of *K* at which the phase transition takes place when there is an intermediate state.

The three master equations can be reduced to a pair taking advantage of the constraint *p*_*x*_ + *p*_*y*_ + *p*_*z*_ = 1. Let us introduce the new variables


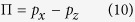


and


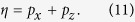


By definition, when the number of individuals in the population is infinite the mean field becomes identical to a probability difference,


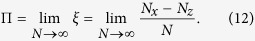


With the transition rates defined by Eqs. (6) (7) (8) and (9), and since Π and *ξ* can be freely interchanged under the infinite *N* condition, the master equations become


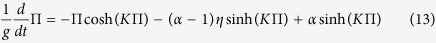



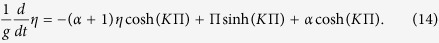


The equilibrium mean field, Π_*eq*_, can be calculated from the master equations by setting the left-hand side of Eqs. (13) and (14) to zero, which leads to the transcendental equation


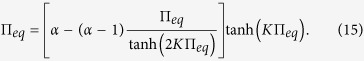


The onset of the phase transition is associated with the mean field departing from zero. Assuming a small but nonvanishing mean field, an expansion of Eq. (15) about Π_*eq*_ = 0 leads to


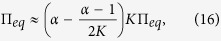


meaning that the critical point is


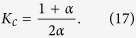


**Figure 1:**
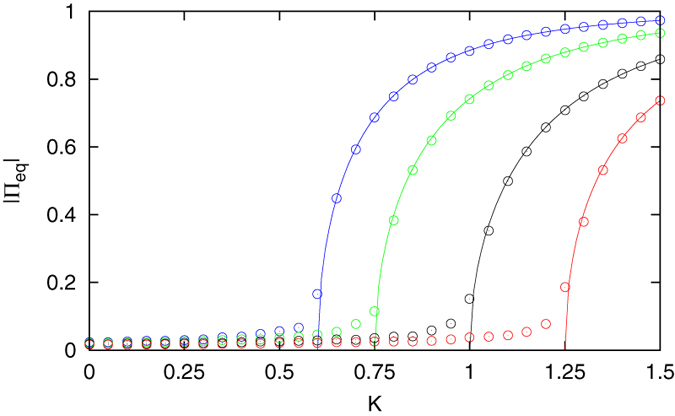
Phase transition in the three-state opinion model. The location of the critical point depends on how fast transitions out of the intermediate state are, which is controlled by the parameter *α*. When *α* = 1 (black) the critical point is at *K*_*c*_ = 1 as in the absence of an intermediate state. Faster transitions out of the middle state, *α* = 2 (green) and *α* = 5 (blue), allow for consensus to be reached with less cooperation. Slower transitions, *α* = 2/3 (red), require more cooperation. The analytical prediction (lines) given by the solution to Eq. (15) agrees with numerical simulation of the model (circles) with *N* = 1000 and *g* = 0.01. The nonvanishing values for the simulation in the disordered regime are due to finite-size fluctuations.

Note that the equilibrium behavior of the two-state decision making model is described by Π_*eq*_ = tanh(*K*Π_*eq*_)^38^, which is identical to the (Curie-Weiss) Ising model^7^. The critical cooperation level is *K*_*c*_ = 1 in the two-state model. These results are recovered by the three-state model if *α* = 1. However, if there is a separation of time scales between transitions into and out of the intermediate state (*α* ≠ 1), the critical point departs from unity. To illustrate this behavior we solve Eq. (15) numerically to find the equilibrium mean field as a function of the cooperation level. Due to symmetry, in the supercritical regime the solution yields two stable equilibrium values with equal magnitude and opposite sign, so we use |Π_*eq*_| as the order parameter which measures the level of consensus while disregarding what direction the global opinion is in. The result is shown in Fig. 1, where for the purpose of comparison we also perform numerical simulations of the actual model with *N* = 1000 individuals and the base transition rate *g* = 0.01. More details on the model simulation procedure can be found in the methods section. As predicted by Eq. (17), if the transition rates out of the middle state are faster than the transition rates into the middle state (*α* > 1), the group is able to form a majority opinion with less cooperative effort, i.e. *K*_*c*_ decreases. In fact, when *α* ≫ 1 only half the effort is required compared to the two-state model. On the other hand, if individuals spend more time undecided (*α* < 1), then more effort is required for the social group to reach consensus compared to the two-state case. Evidently an intermediate, unbiased state can be advantageous to the formation of collective opinions within a social group only when individuals are undecided for relatively short intervals.

The benefit of the middle state to consensus under the condition *α* > 1 only exists when the cooperation level surpasses the threshold 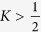. This becomes clear by considering *α* as the control parameter while setting a constant value for *K*. According to the prior analysis for *K*, we expect a phase transition when moving from *α* = 0, where Π_*eq*_ = 0 because all individuals are undecided, toward the limit *α* = ∞, if the cooperation level is high enough. Rewriting Eq. (17), the critical value of *α* is


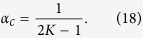


For a fixed value of *K* such that 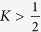, a phase transition from disorder to order occurs when *α* is increased beyond the critical point *α*_*c*_ given in Eq. (18). However, *α*_*c*_ goes to infinity as *K* approaches 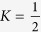, and then becomes negative when 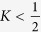. This indicates that for low cooperation levels, 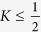, the phase transition no longer occurs and consensus is not possible.

To get more insight into how the middle state affects consensus we can define an effective two-state model from the equilibrium mean field behavior expressed in Eq. (15). We set the condition


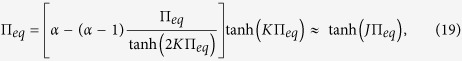


where *J* = *J*(*α*, *K*) is the effective cooperation level of the two-state model that approximates the original three-state model. Expanding Eq. (19) out to the first order in Π_*eq*_ we find


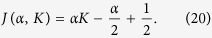


The three-state model, with two control parameters *α* and *K*, can be roughly approximated by a two-state model with one control parameter *J*(*α*, *K*) given by Eq. (20).

**Figure 2:**
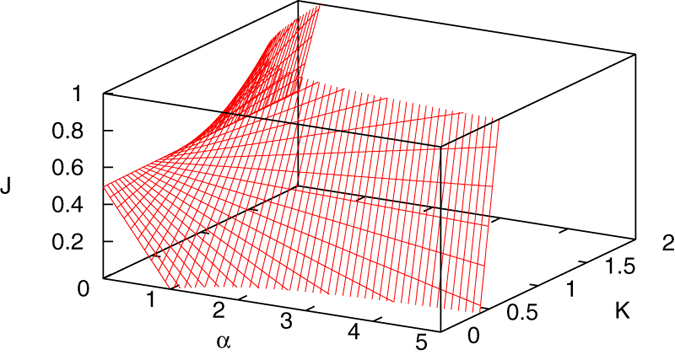
Effective cooperation level in a two-state approximation of the three-state model. The shaded surface represents *J*(*α*, *K*) in Eq. (20) for values of *J* limited to the region [0, 1]. Consensus is possible when *J* > 1. The curve formed by the intersection of *J*(*α*, *K*) with the *J* = 1 plane marks the phase transition boundary defined in Eq. (17) and Eq. (18).

Figure 2 illustrates how the effective cooperation level behaves for different values of the three-state model parameters. The nonlinear coupling between *α* and *K*, represented in the first term on the right-hand side of Eq. (20), leads to non-trivial behavior for *J*. Recall that in the two-state model *J* = 1 marks the critical point between disorder (*J* < 1) and order (*J* > 1). Looking at Fig. 2, we see that the phase transition in the three-state model is defined by a curve lying in the *J* = 1 plane, which is an alternative way to visually represent Eq. (17) and Eq. (18). Additionally, we see that increasing *α* when 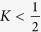 results in lower effective cooperation, and even negative values. However, for cooperation levels 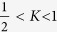, where without the middle state the model would be in the disordered regime, increasing *α* makes consensus emerge in the three-state model.

### Impact of zealots

With the phase transition behavior of the model characterized, we now turn our attention to the inclusion of zealots among the ordinarily functioning individuals. It is known that a few committed individuals can tip the scales in favor of their opinion during a decision-making process^17^,^25^,^28^,^29^,^30^. Our goal here is to understand how the persuasive power of the zealots affects the opinion formation dynamics of the rest of the population. We introduce a total of *m* zealots into the population while *n* individuals remain free to change opinion, so that


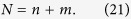


The mean field of the free individuals is


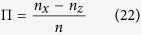


whereas the mean field of the entire population is


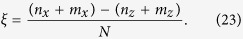


We denote the fraction of zealots in the population by


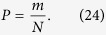


Then the mean field of the entire population becomes


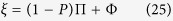


with


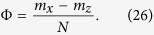


Following the same prescription as before, the master equations describing the dynamics of the free individuals in the presence of the zealots can be written as


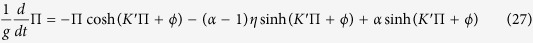



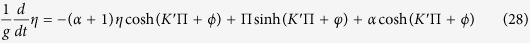


with


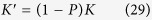


and


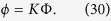


Note how the mean field of the entire population, Eq. (25), appears in the arguments of the hyperbolic functions in Eqs. (27) and (28). This is because a free individual, making transitions according to the rates in Eqs. (6) (7) (8) and (9), now perceives the zealots in addition to the other free individuals.

The equilibrium mean field of the free individuals in the presence of zealots is determined by


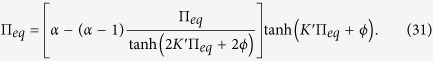


**Figure 3:**
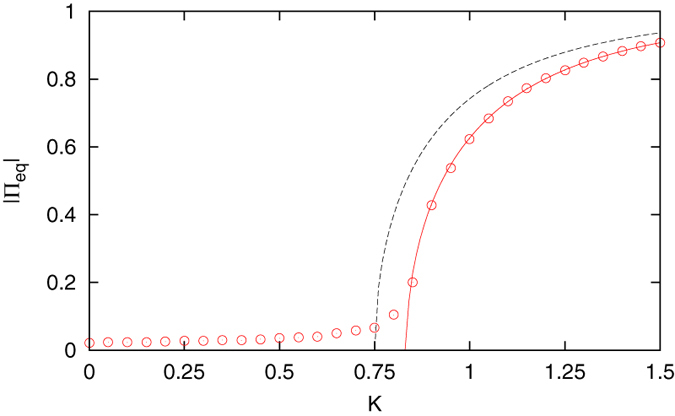
Phase transition behavior when *P* = 0.10 zealots are in the intermediate state and *α* = 2. Compared to the absence of zealots (black, dashed line), more cooperation is required to reach consensus when all the zealots occupy the middle state (red, solid line). Simulations of the model (circles), with *N* = 1000, *m*_*y*_ = 100 and *g* = 0.01, support the analytical prediction.

We solve Eq. (31) numerically to study the impact of zealots on the phase transition behavior. First we consider the unbiased case Φ = 0, and in particular, locate all the zealots in the intermediate state. The result is illustrated in Fig. 3.

When zealots occupy the intermediate state, the phase transition occurs at a higher cooperation level as described by the effective control parameter in Eq. (29). Specifically in the case shown, where *α* = 2 and *P* = 0.10, the critical point has shifted from *K*_*c*_ = 0.75 to *K*_*c*_ = 0.83. In general we can use


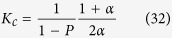


to find the location of the critical point when there are an equal number of zealots in the polar states *x* and *z*. As briefly mentioned before, at the critical point the single equilibrium value at Π_*eq*_ = 0 splits into three branches. The value Π_*eq*_ = 0 becomes unstable for *K* > *K*_*c*_, and the symmetric nonzero branches indicate equal and opposite stable equilibrium states with the majority of free individuals in either *x* or *z*. As long as there are an equal number of zealots in *x* and *z*, this pitchfork bifurcation is preserved, and the relevant information about the phase transition is still captured by the order parameter |Π_*eq*_|.

**Figure 4:**
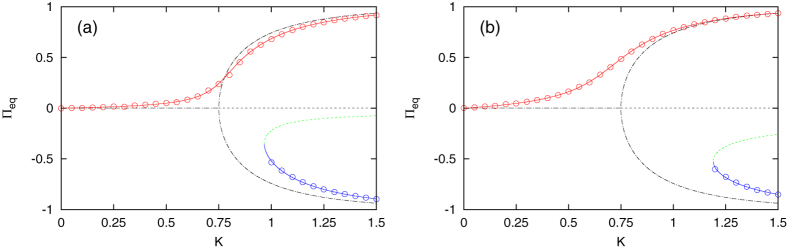
Equilibrium mean field behavior when zealots favor the state *x* and *α* = 2. The disorder-order transition becomes less abrupt as more zealots occupy the state *x*, Φ = 0.03 (**a**) and Φ = 0.10 (**b**). The zealots create a bias in the global opinion toward *x*, destroying the symmetry of the model dynamics in the absence of zealots (black, dash-dot lines). A stable majority in *x* (red, solid line) exists for all values of *K*, while a stable majority in *z* (blue, solid line) exists only for high *K* values. The unstable equilibrium (green, dashed line) dividing the two regimes is shifted to negative values. The analytical prediction (lines) given by the solution to Eq. (31) agrees with numerical simulation of the model (circles) with *N* = 1000, *g* = 0.01, and the values (*m*_*x*_, *m*_*y*_, *m*_*z*_) set to (50, 30, 20) in (**a**) and (100, 0, 0) in (**b**).

Next we consider the case where zealots favor one of the polar opinions, such that Φ ≠ 0. We focus only on Φ > 0 (a majority of zealots in the state *x*), noting that an analogous description applies for Φ → −Φ. Setting *α* = 2 and *P* = 0.10, we evaluated the equilibrium mean field behavior under two different conditions for how the zealots were distributed between the three states: Φ = 0.03 and Φ = 0.10. The results are depicted in Fig. 4. We no longer use the order parameter |Π_*eq*_|, but instead look at the complete equilibrium behavior. That is because the imbalance of zealots disrupts the symmetry between *x* and *z*, which can be seen in Fig. 4 when comparing to the equilibrium behavior in the absence of zealots. The zealot majority in state *x* allows the free individuals to begin to form a consensus in that direction with less cooperation; the onset of the disorder-order transition is gradual instead of abrupt. Relating back to the Ising model, zealots act like an external magnetic field. A critical point can no longer be defined since biased zealots create a nonvanishing mean field among the rest of the population even when the control parameter *K* is arbitrarily small.

Furthermore, it takes a considerable amount of cooperation before the existence of a majority opinion in the state *z* not favored by zealots can be established. These effects become more pronounced as the zealot majority is increased from Φ = 0.03 to Φ = 0.10. The majority of zealots in *x* will eventually overpower a majority of free individuals in *z* if the latter is not great enough to surpass the unstable equilibrium branch. This conversion can happen even if the majority of zealots is weaker than the opposing majority of free individuals. When Φ = 0.03 and *K* = 0.97 for instance, the small zealot majority is enough to convert to state *x* an opposing free individual majority of up to an order of magnitude more, Π = −0.30. Numerical simulations of the model dynamics verify the analytical result of Eq. (31).

## Discussion

Modeling indecision with an intermediate state reveals important information about the dynamics of opinion formation. The critical point at which consensus becomes possible is no longer static with respect to the network structure like it would be in a two-state model. Instead, the location of the critical point is dynamically controlled by the time scale separation between transitions into and out of the middle state. Counterintuitively, giving individuals the ability to refrain from picking a side can make it easier for a group to reach consensus. That is, however, only if the duration of indecision is relatively short-lived. In fact, the adverse effect will occur if individuals tend to remain undecided rather than select an opinion. Regardless of how fast individuals transition from the middle state, when the cooperation level between individuals is too low there is no phase transition and consensus cannot be reached.

Zealots can make it more difficult for the rest of the individuals to reach consensus, even if they do not bias the global opinion in any direction. When zealots do favor one opinion over the other, the abrupt nature of the phase transition is lost, and a matching global opinion forms among free individuals to some degree for any amount of cooperation. A high level of cooperation among free individuals is needed in order to form a stable majority opinion in opposition to a small but organized group of zealots.

The varied effects of the middle state in consensus formation, with or without zealots, seem to elude a simple and intuitive physical explanation using the critical point analysis as we have done. A more complete analysis of the model using a Langevin or Fokker-Planck approach would be helpful to better understand the effects of the middle state, and at the same time would allow for finite-size fluctuations. Such an analysis has been carried out for other three-state models, particularly those with absorbing states similar to the voter model^39^,^40^. The Ising-like model we consider does not have absorbing states and it would be interesting to compare between the two model classes. Additionally it may be useful to coarse-grain time such that direct transitions between the polar states become possible, with the middle state acting to slow these transitions. The coarse-grained transition rates may provide insight into the middle state behavior complementary to the effective cooperation level we defined by approximating the equilibrium mean field behavior of the three-state model with a two-state model.

Increasing the number of intermediate states in the model is expected to lead to consensus with less cooperative effort required. A preliminary study of a four-state model, where there are two unbiased intermediate states, supports this conjecture. Whether or not consensus can be reached with an arbitrarily small amount of cooperation between agents in a model containing many successive intermediate states remains an intriguing open question.

## Methods

The numerical procedure used to simulate the model dynamics is as follows. We fix the number of individuals *N*, the base transition rate *g*, the factor *α*, and the cooperation level *K*. Specifically, we used the values of *N* = 1000 and *g* = 0.01 in all simulations. The system is prepared in an initial state having a vanishing mean field by placing half of the units in *y*, one quarter in *x* and one quarter in *z*. For convenience we set the computational time step to Δ*t* = 1. At each time step the units have a chance to switch states. The value of the mean field at the previous time is used to calculate the transition rates, Eqs. (6) (7) (8) and (9). Then the transition rates are converted to transition probabilities through multiplication by Δ*t*. Note that since Δ*t* = 1 we set *g* ≪ 1 in order to ensure that the conversion of the transition rates into probabilities is valid. A uniform random number is drawn from the interval [0, 1) for each individual in the middle state to determine the direction of the possible transition. If the random number is less than 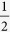 the corresponding individual will attempt to make a transition to *x*, and otherwise to *z*. Then for every individual, a uniform random number is drawn from the interval [0, 1) to determine if a transition takes place. A transition occurs if the random number is less than the transition probability, otherwise the individual keeps its state. This process is repeated to generate the trajectories *s*_*i*_(*t*) of the individuals for the desired number of time steps.

The order parameter values |Π_*eq*_| in Figs 1 and 3 were calculated through a time average of the absolute value of the mean field. The equilibrium mean field values in Fig. 4 were calculated through a time average of the mean field, with an initial preparation of *ξ*(0) = 0 for the positive branch, and *ξ*(0) = −1 for the negative branch. In nearly all cases the time average was taken over the second half of a trajectory having *T* = 2 × 10^5^ time steps, the exception being the following. In Fig. 4, for the minimum cooperation level at which the negative equilibrium branch first appears, *K* = 1.0 in panel (a) and *K* = 1.2 in panel (b), the negative equilibrium point is not stable enough to yield a consistent time-averaged value. The barrier centered at the unstable equilibrium point is weak enough to be surpassed by the finite-size fluctuations, and a long time average over the mean field will yield the positive equilibrium value regardless of the initial preparation. For this reason a shorter time duration was adopted when generating these two data points.

## Additional Information

**How to cite this article**: Svenkeson, A. and Swami, A. Reaching Consensus by Allowing Moments of Indecision. *Sci. Rep.* **5**, 14839; doi: 10.1038/srep14839 (2015).
